# Rab27A promotes cellular apoptosis and ROS production by regulating the miRNA‐124‐3p/STAT3/RelA signalling pathway in ulcerative colitis

**DOI:** 10.1111/jcmm.15726

**Published:** 2020-08-20

**Authors:** Yang Luo, Min‐Hao Yu, Ya‐Ru Yan, Yong Zhou, Shao‐Lan Qin, Yi‐Zhou Huang, Jun Qin, Ming Zhong

**Affiliations:** ^1^ Department of Gastrointestinal Surgery Renji Hospital School of Medicine Shanghai Jiao Tong University Shanghai P.R. China; ^2^ Department of Respiratory and Critical Care Medicine Ruijin Hospital Shanghai Jiao Tong University School of Medicine Shanghai China; ^3^ Department of Gastrointestinal Surgery Jiading Hospital of Traditional Chinese Medicine Shanghai China

**Keywords:** DSS‐induced colitis model, miR‐124‐3p, Rab27A, STAT3/RelA signalling pathway, ulcerative colitis

## Abstract

Ulcerative colitis (UC) is a multifactorial inflammatory disease, and increasing evidence has demonstrated that the mechanism of UC pathogenesis is associated with excessive cellular apoptosis and reactive oxygen species (ROS) production. However, their function and molecular mechanisms related to UC remain unknown. In this study, Rab27A mRNA and protein were proven to be overexpressed in intestinal epithelial cells of UC patients and DSS‐induced colitis mice, compared with control (*P* < 0.05). And Rab27A silencing inhibits inflammatory process in DSS‐induced colitis mice (*P* < 0.05). Then, it was shown that knockdown of Rab27A suppressed apoptosis and ROS production through modulation of miR‐124‐3p, whereas overexpression of Rab27A promoted apoptosis and ROS production in LPS‑induced colonic cells. In addition, enhanced expression of miR‐124‐3p attenuated apoptosis and ROS production by targeting regulation of STAT3 in LPS‑induced colonic cells. Mechanistically, we found Rab27A reduced the expression and activity of miR‐124‐3p to activate STAT3/RelA signalling pathway and promote apoptosis and ROS production in LPS‑induced colonic cells, whereas overexpression of miR‐124‐3p abrogated these effects of Rab27A. More importantly, animal experiments illustrated that ectopic expression of Rab27A promoted the inflammatory process, whereas overexpression of miR‐124‐3p might interfere with the inflammatory effect in DSS‐induced colitis mice. In summary, Rab27A might modulate the miR‐124‐3p/STAT3/RelA axis to promote apoptosis and ROS production in inflammatory colonic cells, suggesting that Rab27A as a novel therapeutic target for the prevention and treatment of UC patients.

**Abbreviations**
DSS: dextran sodium sulphate
IBD: inflammatory bowel disease
IECs: intestinal epithelial cells
RT‐qPCR: RNA isolation and real‐time quantitative PCR
UC: ulcerative colitis

## 1 INTRODUCTION

Ulcerative colitis (UC) is a chronic inflammatory bowel disease (IBD) that affects both children and adults, and is characterized by periods of relapse followed by periods of remission.^1^ Because of the westernized dietary lifestyle, the incidence of UC in several Asian countries, especially in China, is rising rapidly.^2^, ^3^ However, although dysregulation in intestinal epithelial cells, such as TNF‐α and IL‐1β,^4^, ^5^ has been widely reported in UC, the molecular basis and pathophysiology of UC are not completely understood.

MicroRNAs (miRNAs) are small non‐coding RNAs that bind to corresponding sequences in the 3'‐untranslated region (UTR) of reciprocal objective mRNAs, thus inhibiting the synthesis of proteins.^6^ Increasing evidence suggests that miRNAs are widely dysregulated in UC, potentially impacting UC pathogenesis, diagnosis and therapy.^6^, ^7^, ^8^ For example, Wu et al found that miR‐206 regulates TNF‐α and IL‐8 in active human UC and dextran sodium sulphate (DSS)‐induced experimental colitis in mice.^8^ Min et al illustrated that miR‐155 overexpression enhanced UC inflammatory activity by down‐regulating the expression of FOXO3a, a key inhibitor of the NF‐κB signalling pathways.^7^

In the past decade, a number of Rab proteins have been demonstrated to be involved in the initiation, development and progression of IBD, such as Rab7b, Rab11 and Rab13.^9^, ^10^, ^11^ Rab27A, belonging to the Ras superfamily of monomeric G proteins, localizes to distinct cellular compartments and regulates specific steps of intracellular membrane trafficking.^12^ Recently, Tang et al showed that Rab27A could directly bind to miR‐124‐3p to inhibit tumorigenesis in osteoclastogenesis.^13^ Although Rab27A has been reported to be up‐regulated in clinical UC patients,^14^ its function and molecular mechanisms related to UC remain unknown. In the present study, we observed that Rab27A mRNA and protein expression levels were increased in both human UC patients and DSS‐induced colitis mice. Subsequent results demonstrated that knockdown of Rab27A suppressed cellular apoptosis and reactive oxygen species (ROS) production in colonic inflammatory cells. Mechanistically, Rab27A could regulate the miR‐124‐3p/STAT3/RelA axis to promote apoptosis and ROS production in ulcerative colitis.

## 2 MATERIALS AND METHODS

### 2.1 Human specimens

Tissue biopsy samples were collected from 12 UC patients and 12 healthy patients undergoing screening colonoscopies or surgery from January 2017 to April 2018 at Renji Hospital, Shanghai, China, after obtaining informed consent. The protocol was approved by the local ethics committee of Renji Hospital, Shanghai, China. The related clinical data are shown in Table 1. The diagnosis of UC was based on standard clinical characteristic and histological criteria, colonoscopy feature, and pathological results.^15^ The patients were obtained before initiation of anti‐inflammatory treatment. The samples were embedded in paraffin for immunofluorescence analysis or immediately used for RT‐qPCR or Western blotting.

**TABLE 1:** The patient of Ulcerative colitis is clinical features

	Ulcerative colitis
No. of patients	12
Age, mean ± SD (yrs)	32.33 ± 1.77
Gender (Male/Female)	7/5
Mean duration of disease (months)	16.25 ± 2.32
Treatment	
surgery	3
No surgery	9

### 2.2 Animal studies

The animal research was approved by the local ethics committee of Renji Hospital, Shanghai, China. Male C57BL/6 mice [age 7‐8 weeks old, weight 20‐22 g] were regularly treated with 2.5% dextran sulphate sodium (DSS, MW 40‐50 kDa; MP Biomedicals, USA) in drinking water for 1 month, after which intestinal mucosa was harvested for further analysis. The severity of colitis was scored by recording standard parameters, including colonic length, inflammatory cell infiltration and histological score. To evaluate the function of Rab27A, the C57BL/6 mice were intracolonically administered 40 μg of Lv‐shRab27A or a control Lv‐shRNA on 1 and 15 days using the previously reported.^16^ Briefly, the appropriate amount of Lv‐shRab27A and its control were resuspended in 100 μL of Opti‐MEM with 2 μL of Lipofectamine 3000.

### 2.3 Histological analysis

The human/mice colonic tissue was stained with H&E, and histological score was blindly established by per Obermeier et al^17^ The intestinal mucosal damage was graded on the following 0‐4 scale: 0—none; 1—minimal loss of goblet cells; 2—extensive loss of goblet cells; 3—minimal loss of crypts and extensive loss of goblet cells; and 4—extensive loss of crypts. Inflammatory infiltration was graded on the following 0‐4 scale: 0—none; 1—infiltrate around crypt bases; 2—infiltrate in muscularis mucosa; 3—extensive infiltrate in muscularis mucosa, with oedema; and 4—infiltration of the submucosa. The histological activity index (HAI) was designated as the sum of the mucosa and infiltration scores, resulting in the total HAI score ranging from 0 (unaffected) to 8 (severe colitis).

### 2.4 Isolation of intestinal epithelial cells (IECs)

The mucosa was collected from human/mice intestines at 4°C and immediately stored at −80°C. The frozen mucosal tissues were homogenized with an OmniTH homogenizer (Beijing Pioneer Trading Co., Ltd., China) at homogenization buffer (50 mmol\L Tris‐HCl, pH 7.2) containing Na_3_VO_4_ and a protease inhibitor cocktail (Sigma‐Aldrich, USA). After ultrasonic treatment, the homogenate was centrifuged at 2500 × g for 5 min. The above supernatant was isolated as total intestinal epithelial proteins, and protein concentrations then were measured by a Bio‐Rad Protein Assay (Hercules, CA, USA).

### 2.5 Cell lines and plasmid transfection

The HT‐29 and Caco‐2 colonic cell lines were obtained from the Cell Bank of the Chinese Academy of Sciences (Shanghai, China). MiR‐124‐3p mimics, miR‐96‐5p mimics, miR‐124‐3p inhibitors and miR‐96‐5p inhibitors were purchased from GenePharma (Shanghai, China), and the sequences are shown in Table S1. Knockdown or overexpression lentivirus vectors (Lv‐shRab27A, Lv‐shSTAT3, Lv‐Rab27A and Lv‐STAT3) were purchased from GenePharma (Shanghai, China). The cells were distributed in 6‐well plates to approximately 50%‐70% confluence and were transfected the next day with plasmid at a concentration of 100 nmol\L in DMEM (GenePharma, Shanghai, China) using Lipofectamine 3000 (Invitrogen, USA), according to the manufacturer's instructions.

### 2.6 RNA isolation and real‐time quantitative PCR (RT‐qPCR)

According to the manufacturer's instructions, total RNA from cell lines and tissue samples was extracted using the TRIzol reagent (Invitrogen, USA). cDNA was synthesized using a microRNA Reverse Transcription Kit (Promega, USA) or a PrimeScript RT‐PCR Kit (Takara, Japan). RT‐qPCR was performed using a StepOne Real‐Time PCR System (Applied Biosystems, USA). Primers for miR‐124‐3p, miR‐96‐5p and U6 were obtained from GeneCopoeia (California, USA). The PCR primer sequences used in this study were shown in Table 2.

**TABLE 2:** Quantitative Real‐time PCR primers used in this study

Gene name	Forward sequence (5’ to3’)	Reverse sequence (5’ to 3’)
Human Rab27A	AGTTGATGGAGCGAACTGCT	CCCTACACCAGAGTCTCCCA
Human STAT3	GGAGAAACAGGATGGCCCAA	ACATCCTGAAGGTGCTGCTC
Human GAPDH	AATGGGCAGCCGTTAGGAAA	GCGCCCAATACGACCAAATC
Human MiR‐96‐5p	TTTGGCACTAGCACATTTTTGCT	mRQ 3’ Primer
Human MiR‐124‐3p	TAAGGCACGCGGTGAATGCC	mRQ 3’ Primer
Human U6	CTCGCTTCGGCAGCACA	mRQ 3’ Primer
Mouse Rab27A	GTGGGGCCAGACGGAAAATA	GTCCTCGCTGTGCTCTATCC
Mouse GAPDH	CCCTTAAGAGGGATGCTGCC	ACTGTGCCGTTGAATTTGCC
Mouse MiR‐124‐3p	GGCATTCACCGCGTGCCTTA	mRQ 3’ Primer
Mouse U6	CTCGCTTCGGCAGCACA	mRQ 3’ Primer

### 2.7 Western blotting

All the proteins were separated by 10% SDS‐PAGE and transferred onto a PVDF membrane (Millipore, Bedford, MA). The membranes were blocked for 90 minutes in TBS containing 0.1% Tween 20 and 5% non‐fat powdered milk and then incubated first with primary antibodies against Rab27A (ab55667, 1:1500, Abcam, UK), STAT3 (ab119352, 1:1500, Abcam, UK), NF‐kB (ab32536, 1:1500, Abcam, UK) or β‐actin (ab8227, 1:1500, Abcam, UK) overnight at 4°C. After incubation with an HRP‐conjugated secondary antibody, specific proteins were visualized with an enhanced chemiluminescence kit (Amersham Corp, Buckinghamshire, UK).

### 2.8 Flow cytometry analysis

Cell apoptosis and ROS production were quantified by flow cytometry analysis (BD Biosciences, USA). IECs were suspended in 1 × binding buffer and incubated with antibodies (BD, Franklin Lakes, USA) to detect apoptosis cells. We use blank control to distinguish autofluorescence and specific fluorescence of cells, FITC‐Annexin V tube or PI tube (single‐positive tube) to adjust fluorescent compensation, and FITC‐Annexin V/PI double positive tube to detect apoptosis cells. Intracellular ROS was labelled by DCFH‐DA (Beyotime, Haimen, China). Briefly, 1‐2 × 10^5^ IECs were resuspended with DMEM contained with 10 μmol\L DCFH‐DA probe. Then, the colonic cells were placed in the dark incubated with 10 mmol\L DCFH‐Dam for 20 min at 37°C. The data were collected on an LSR‐Fortessa X20 flow cytometer (BD, Franklin Lakes, USA). Flow cytometry was performed after the cells were washed with PBS three times.

### 2.9 Luciferase activity assay

DNA fragments of putative wild‐type (WT) and mutant (Mut) miR‐124‐3p binding sites in the 3'‐UTR of Rab27A (Rab27A WT: 5'‐UCACCUGCCUUAA‐3', Rab27A Mut: 5'‐AUUUUAUAAAAUU‐3') were cloned into a pmirGLO‐Report luciferase vector (Genearray Biotechnology, China) (Table S2). The reporter plasmid was transfected into HT‐29 and Caco‐2 cells in the presence of either miR‐124‐3p mimics or miR‐96‐5p mimics. After 48 hours transfection period, cells were harvested and then analysed using a Dual Luciferase Assay kit (Promega, USA). Normalized firefly luciferase activity (firefly luciferase activity/Renilla luciferase activity) for each construct was compared with that of the pmirGLO vector group. Each experiment was repeated in triplicates.

### 2.10 Statistical analysis

The statistical differences were analysed using the Student's *t* test between two groups or chi‐squared testing between multiple groups by using SPSS 22.0 statistical package software (SPSS, Chicago, IL). The value of *P* < 0.05 was considered statistically significant.

## 3 RESULTS

### 3.1 Rab27A mRNA and protein expression levels were up‐regulated in IECs from UC patients and experimental animal

**FIGURE 1:**
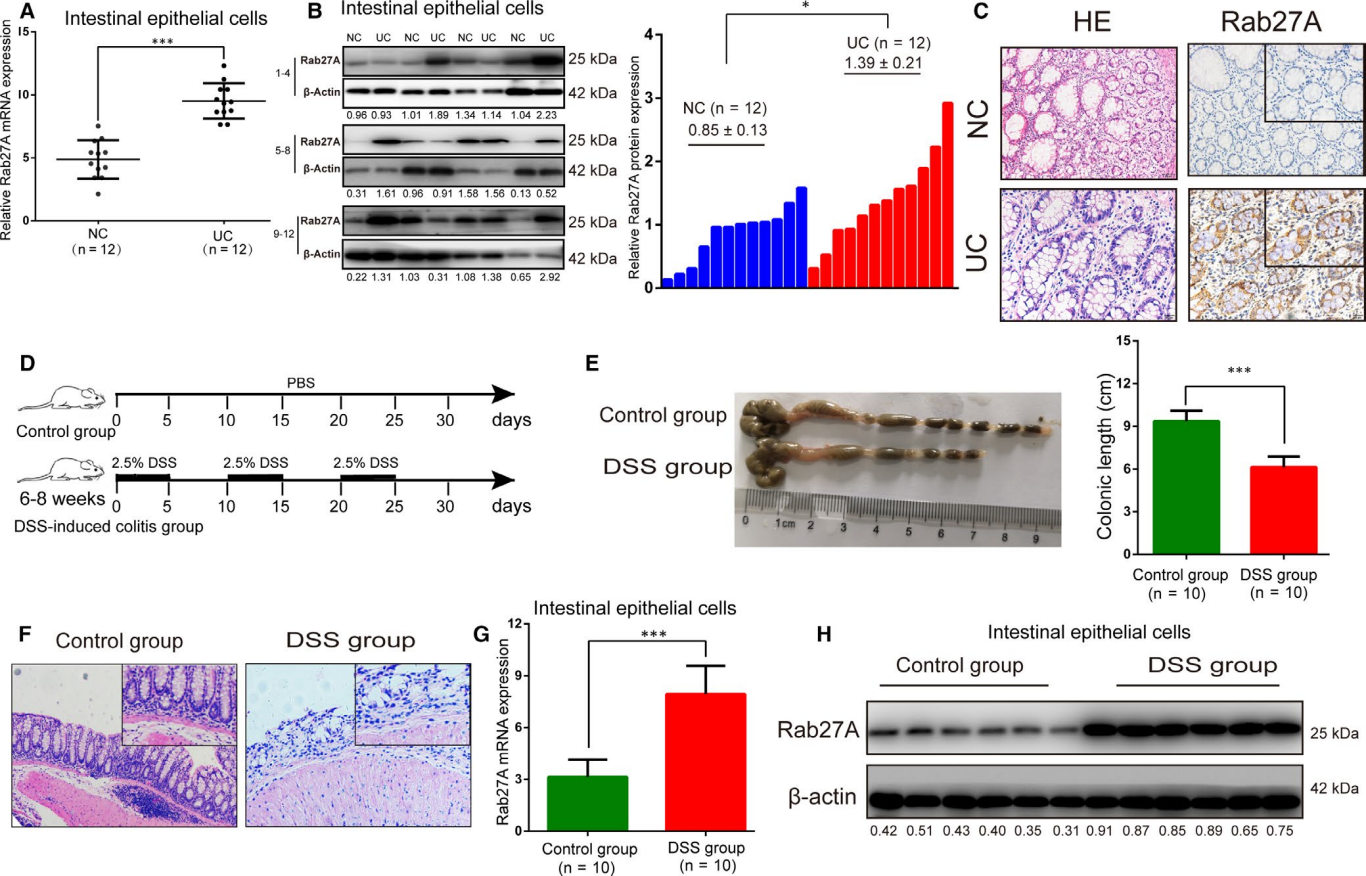
Rab27A expression is markedly increased in IECs from UC tissues. A‐C: Rab27A mRNA and protein expression levels in IECs of UC and normal tissues were determined by RT‐qPCR (A), Western blotting (B) and immunohistochemistry (C). D: Schematic representation of the protocol for the DSS‐induced colitis mouse model in C57BL/6 mice. E‐F: The DSS‐induced colitis mouse model was evaluated by colonic length (E) and H&E staining (F). G‐H: The Rab27A mRNA and protein expression levels in IECs of the DSS‐induced colitis mouse model and control group determined by RT‐qPCR (G) and Western blotting (H). Data are presented as means ± SD of three independent experiments. *P < .05, ***P < .001

To examine the expression status of Rab27A in UC tissues, we isolated IECs from UC patients. Rab27A mRNA and protein expression levels were markedly up‐regulated in UC tissues compared with those in matched normal tissues (Figure 1A,B). Moreover, immunohistochemical analysis revealed that Rab27A protein in the inflamed colonic mucosa of UC tissues was highly expressed, especially in the epithelial layer, compared with that in the control colonic tissue mucosa (Figure 1C). Next, we established a DSS‐induced colitis mouse model that has been used extensively to study the pathogenesis of ulcerative colitis (Figure 1D‐F). As shown in Figure 1G‐H, Rab27A mRNA and protein were extremely overexpressed in IECs of DSS‐induced mice. Taken together, these data indicate that Rab27A is dysregulated in ulcerative colitis.

### 3.2 Knockdown of Rab27A reduced UC progression in inflammatory colonic cells

**FIGURE 2:**
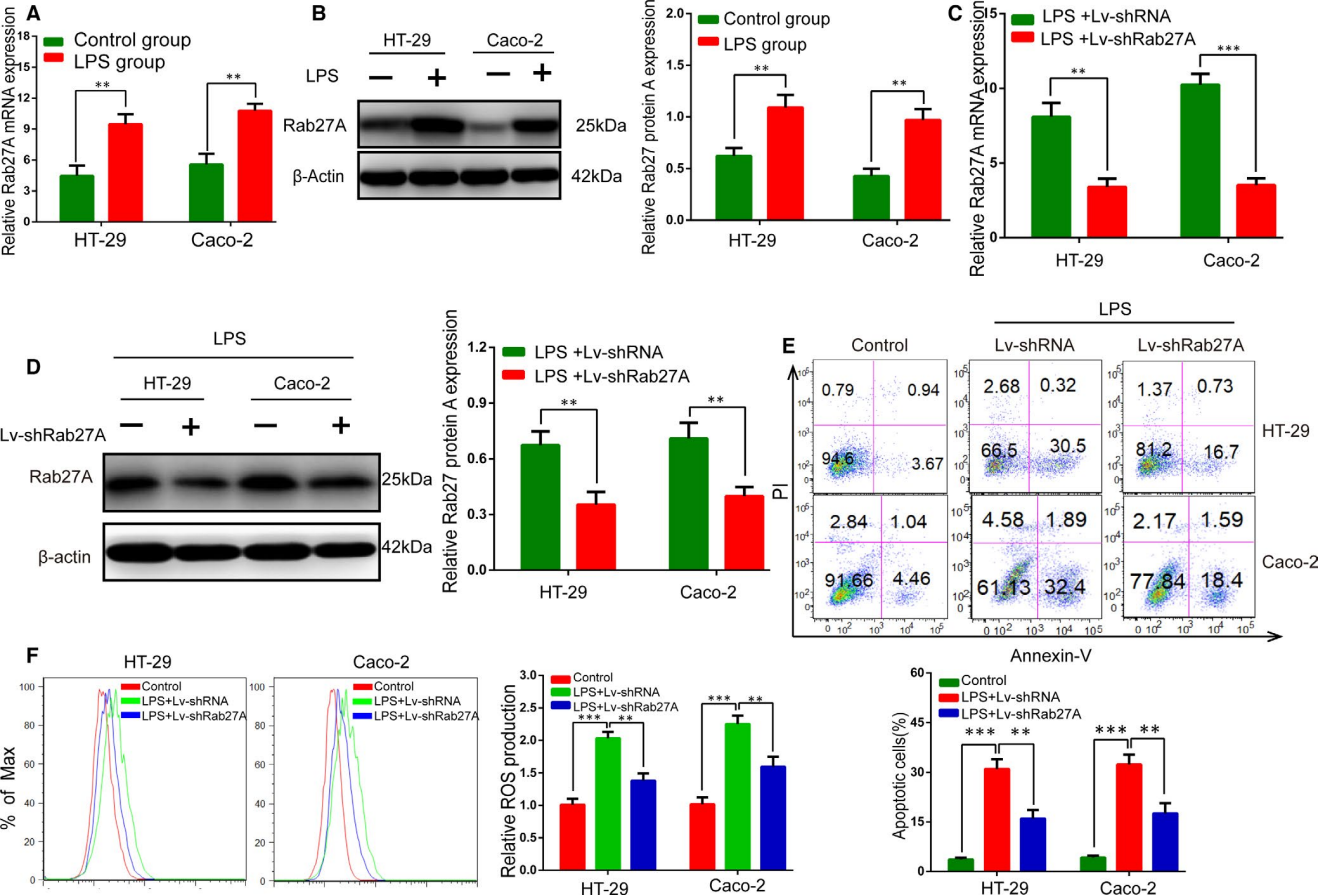
Rab27A inhibition reduces ulcerative colitis progression in LPS‑induced inflammatory colonic cells. A‐B: RT‑qPCR (A) and Western blotting (B) analyses of the relative expression of Rab27A in LPS‑induced HT‑29 and Caco‐2 inflammatory cell lines. C‐D: Rab27A knockdown efficiency was confirmed by RT‐qPCR (C) and Western blotting (D) in LPS‑induced inflammatory cells. E‐F: Cellular apoptosis (E) and ROS production (F) were monitored by flow cytology in LPS‑induced inflammatory cells treated with Lv‑shRab27A or Lv‐shRNA. Data are presented as means ± SD of three independent experiments. **P < .01, ***P < .001

Lipopolysaccharide (LPS), the major outer membrane constituent of Gram‐negative bacteria, stimulates production of pro‐inflammatory cytokines such as IL‐1β and TNF‐α.^18^ To identify the regulatory role of Rab27A in inflammatory colonic cells, HT‑29 and Caco‐2 cells were induced using 10 ng/mL LPS to establish ulcerative colitis cell models, which were confirmed by detecting the inflammatory factors TNF‑α and IL‐1β^19^ (Figure S1). First, Rab27A mRNA and protein expression levels were significant higher in LPS‑induced colonic cells than in those of the control groups (Figure 2A,B). Then, RT‐qPCR and Western blotting verified the effectiveness of the lentiviral vector for Rab27A interference (Figure 2C,D). As a result, Lv‑shRab27A could reduce cellular apoptosis and ROS production, according to flow cytology (Figure 2E,F). Collectively, these results indicated that knockdown of Rab27A might suppress the biological functions of ulcerative colitis.

### 3.3 Knockdown of Rab27A suppressed UC progression in DSS‑induced colitis mice

**FIGURE 3:**
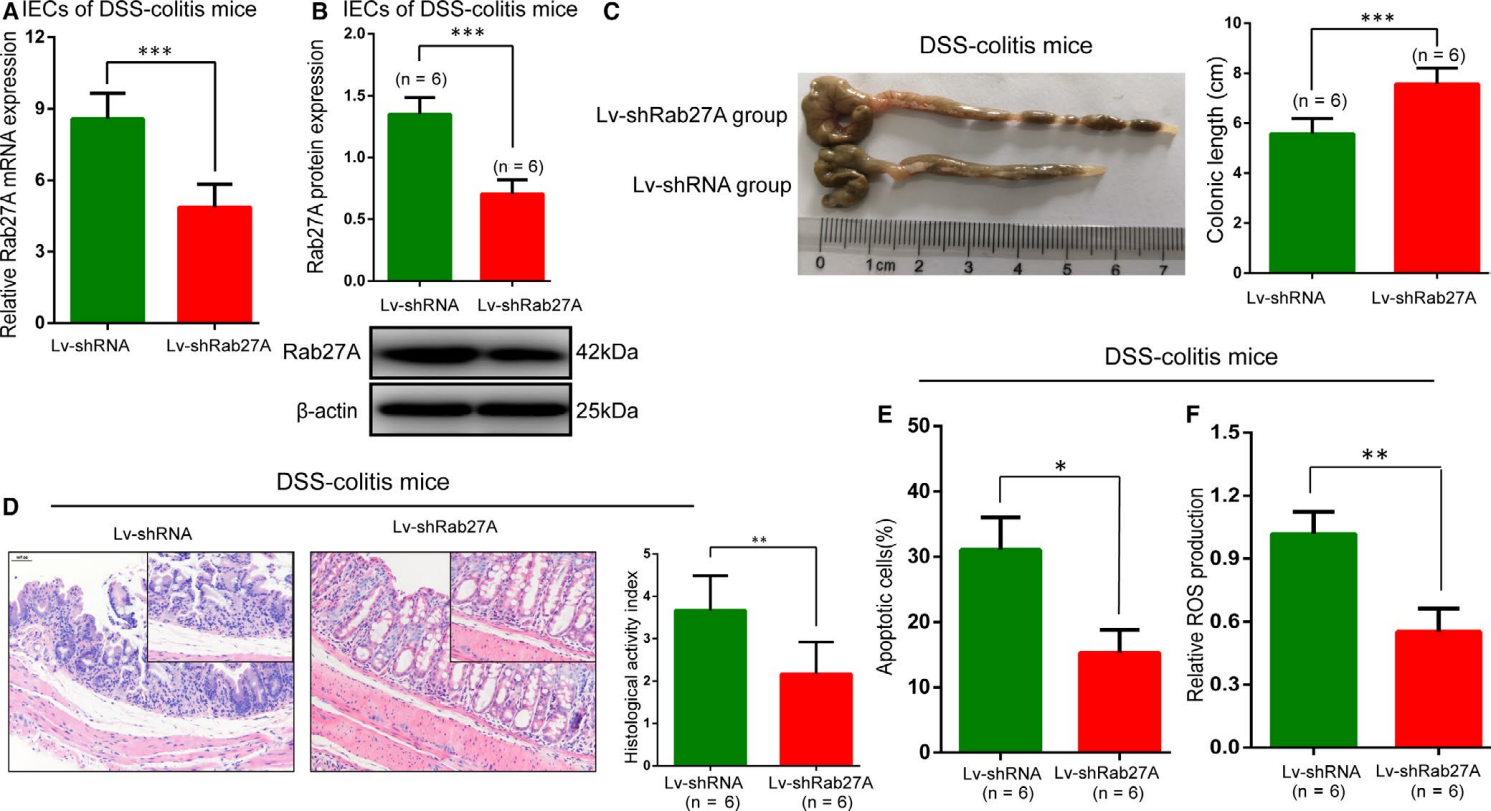
Knockdown of Rab27A suppresses UC progression in DSS‑induced colitis mice. A‐B: Rab27A knockdown efficiency was confirmed by RT‐qPCR (A) and Western blotting (B) in DSS‐induced colitis mice. C‐D: The colonic length (C) and histological score (D) by H&E staining were measured to assess the effects of Lv‑shRab27A or Lv‐shRNA in DSS‐induced colitis mice. E: Cellular apoptosis and ROS production were measured by flow cytology in DSS‐induced colitis mice treated with Lv‑shRab27A or Lv‐shRNA. Data are presented as means ± SD. *P < .05, **P < .01, ***P < .001

To improve our understanding of the functions of Rab27A in UC, we treated DSS‐induced colitis mice with Lv‐shRab27A (Lv‐shRab27A/DSS group), compared with DSS‐induced colitis mice with scrambled shRNA (Lv‐shRNA/DSS group) through intracolonic administration. Rab27A mRNA and protein levels in IECs were reduced in the Lv‐shRab27A/DSS group (Figure 3A,B). The colonic length of the Lv‐shRab27A/DSS group was markedly shorter than that of the Lv‐shRNA/DSS group (Figure 3C). Histological analysis indicated that Lv‐shRab27A repressed monocyte infiltration and intestinal mucosal erosions and produced a lower histological score than Lv‐shRNA (Figure 3D). Furthermore, Lv‑shRab27A could reduce cellular apoptosis (Figure 3E) and ROS production (Figure 3F) in the Lv‐shRab27A/DSS group. Therefore, these results suggested that knockdown of Rab27A might inhibit the pathogenesis of ulcerative colitis.

### 3.4 Rab27A directly interacts with miR‐124‐3p in ulcerative colitis

**FIGURE 4:**
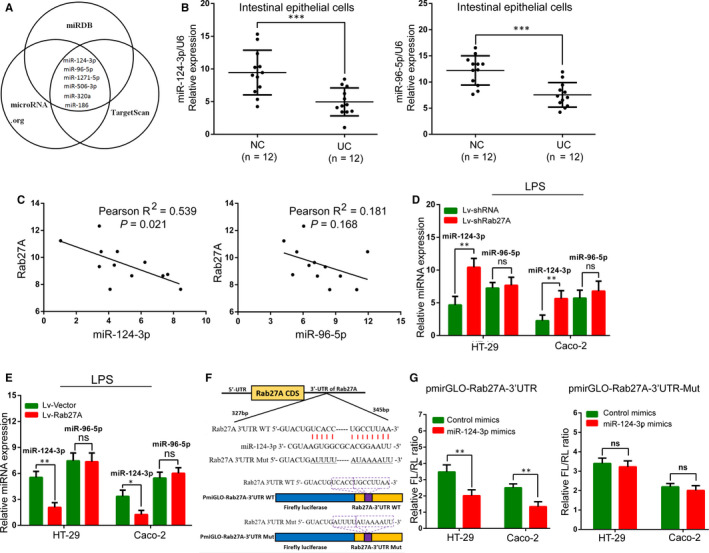
Rab27A directly interacts with miR‐124‐3p in ulcerative colitis. A: Bioinformatic software was used to predict miRNAs potentially targeting Rab27A. B: The levels of miR‐124‐3p and miR‐96‐5p in UC and normal tissues were determined by RT‐qPCR. C: Rab27A expression was inversely correlated with miR‐124‐3p but not miR‐96‐5p levels in UC tissues. D‐E: miR‐124‐3p and miR‐96‐5p expression levels in colonic inflammatory cells in the presence of Lv‐shRab27A (D) or Lv‐Rab27A (E) were detected by RT‐qPCR. F: The miR‐124‐3p target site in the 3’‐UTR of Rab27A mRNA was predicted, and the mutated site in the 3’‐UTR of Rab27A is also shown. G: Luciferase activity was detected after cotransfection of the Rab27A 3’‐UTR or its mutant form and miR‐124‐3p mimics or control mimics in colonic inflammatory cells. Data are presented as means ± SD of three independent experiments. *P < .05, **P < .01, ***P < .001

In recent decades, the knowledge of miRNAs in UC has expanded, indicating that miRNAs play an important role in regulating inflammatory processes.^7^, ^8^ In this study, we have been suggested that miRNA regulated Rab27A expression in UC patients. We used three bioinformatic software programs (miRDB, microRNA.org and TargetScan) to predict miRNAs that could potentially target Rab27A. We identified 6 miRNAs as candidates that could potentially bind to the Rab27A 3'‐UTR (Figure 4A). RT‐qPCR results showed that miR‐124‐3p and miR‐96‐5p were notably down‐regulated in UC tissues (Figure 4B), whereas miR‐506‐3p and miR‐320a were up‐regulated, and the expression of miR‐186 and miR‐1271‐5p observed no changes in UC tissues (Figure S2). Similarly, the levels of Rab27A were found to inversely correlate with those of miR‐124‐3p but not miR‐96‐5p in UC tissues (Figure 4C). Subsequently, we revealed that miR‐124‐3p expression was notably increased by treatment with Lv‐shRab27A and significantly repressed by treatment with Lv‐Rab27A in inflammatory colonic cells, whereas miR‐96‐5p expression observed no changes (Figure 4D‐E). Next, we constructed two luciferase reporter plasmids with either a wild‐type or mutant Rab27A 3'‐UTR (mutant miR‐124‐3p‐binding site) (Figure 4F). The miR‐124‐3p mimics notably suppressed the luciferase reporter activity of the wild‐type Rab27A 3'‐UTR, whereas no significant difference was showed in the activity of the mutant Rab27A 3'‐UTR (Figure 4G). Overall, we speculated that miR‐124‐3p was the regulatory miRNA responsible for Rab27A 3'‐UTR activity in colonic inflammatory cells.

### 3.5 Rab27A regulated UC progression through miR‐124‐3p

**FIGURE 5:**
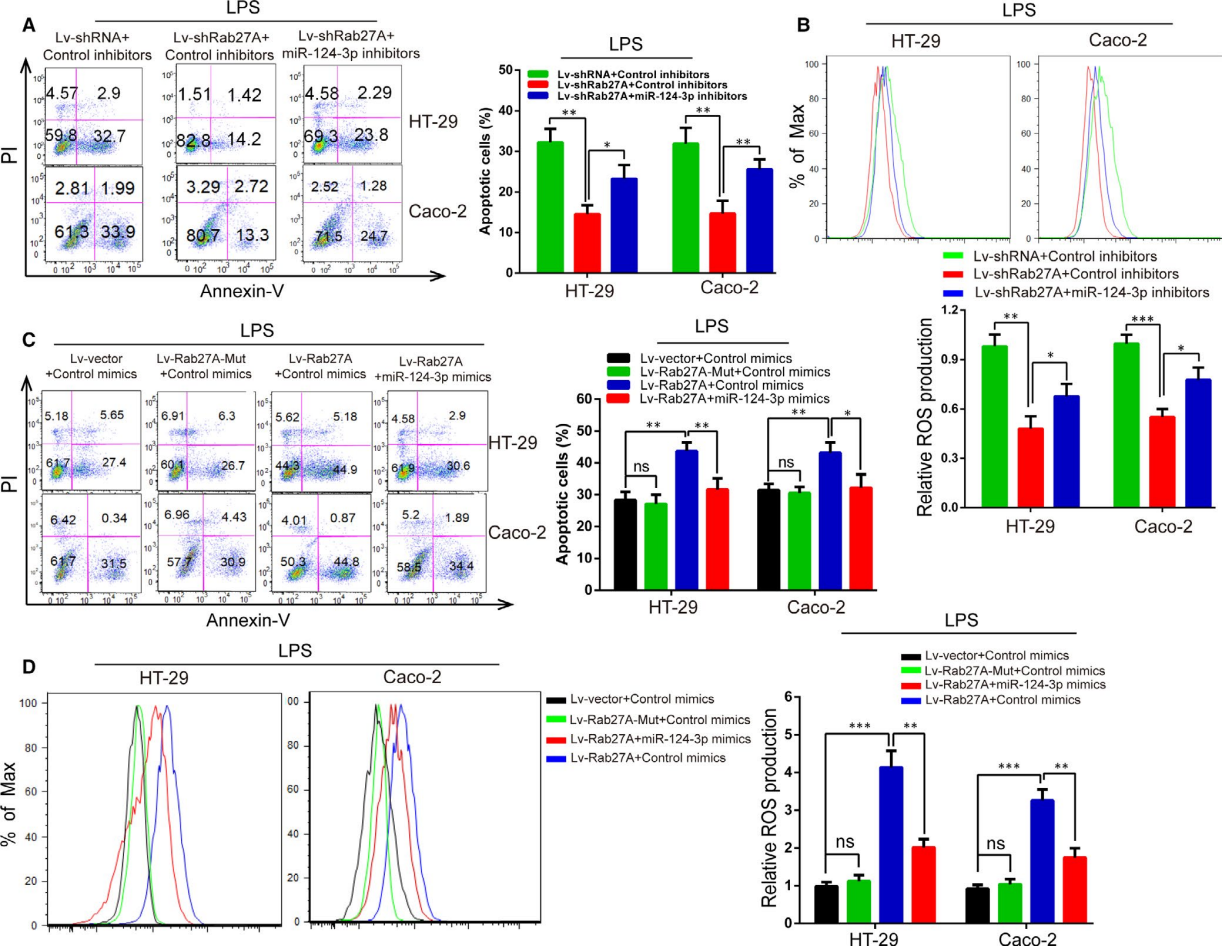
Rab27A regulates UC progression through miR‐124‐3p. A‐B: The effects of apoptosis (A) and ROS production (B) were analysed using flow cytometry in colonic inflammatory cells transfected with Lv‐shRab27A or cotransfected with Lv‐shRab27A and miR‐124‐3p inhibitors. C‐D: The effects of apoptosis (C) and ROS production (D) were analysed using flow cytometry in colonic inflammatory cells transfected with Lv‐Rab27A or Lv‐Rab27A‐Mut or cotransfected with Lv‐Rab27A and miR‐124‐3p mimics. Data are presented as means ± SD of three independent experiments. *P < .05, **P < .01, ***P < .001

To verify whether Rab27A exerts biological functions in LPS‑treated IECs through miR‐124‐3p, rescue experiments were carried out. Flow cytology assays revealed that the down‐regulation of Rab27A greatly inhibited the cellular apoptosis or ROS production, and miR‐124‐3p inhibitors could counteract these effects (Figure 5A,B). In addition, miR‐124‐3p mimics promoted the effect of Lv‐shRab27A, which inhibited the cellular apoptosis or ROS production (Figure S3A,B). What's more, up‐regulation of Rab27A increased the cellular apoptosis and ROS production compared with those of control groups, but increased Lv‐Rab27A‐Mut (mutant miR‐124‐3p‐binding site) expression did not enhance these effects (Figure 5C,D). Intriguingly, miR‐124‐3p mimics reversed these the effects induced by Lv‐Rab27A, which promoted the apoptosis or ROS production (Figure 5C,D). In addition, miR‐124‐3p inhibitors accelerated the effect of Lv‐Rab27A (Figure S3C,D). Therefore, these data indicated that Rab27A promoted UC progression through miR‐124‐3p.

### 3.6 Rab27A regulated STAT3 expression by binding with miR‐124‐3p

**FIGURE 6:**
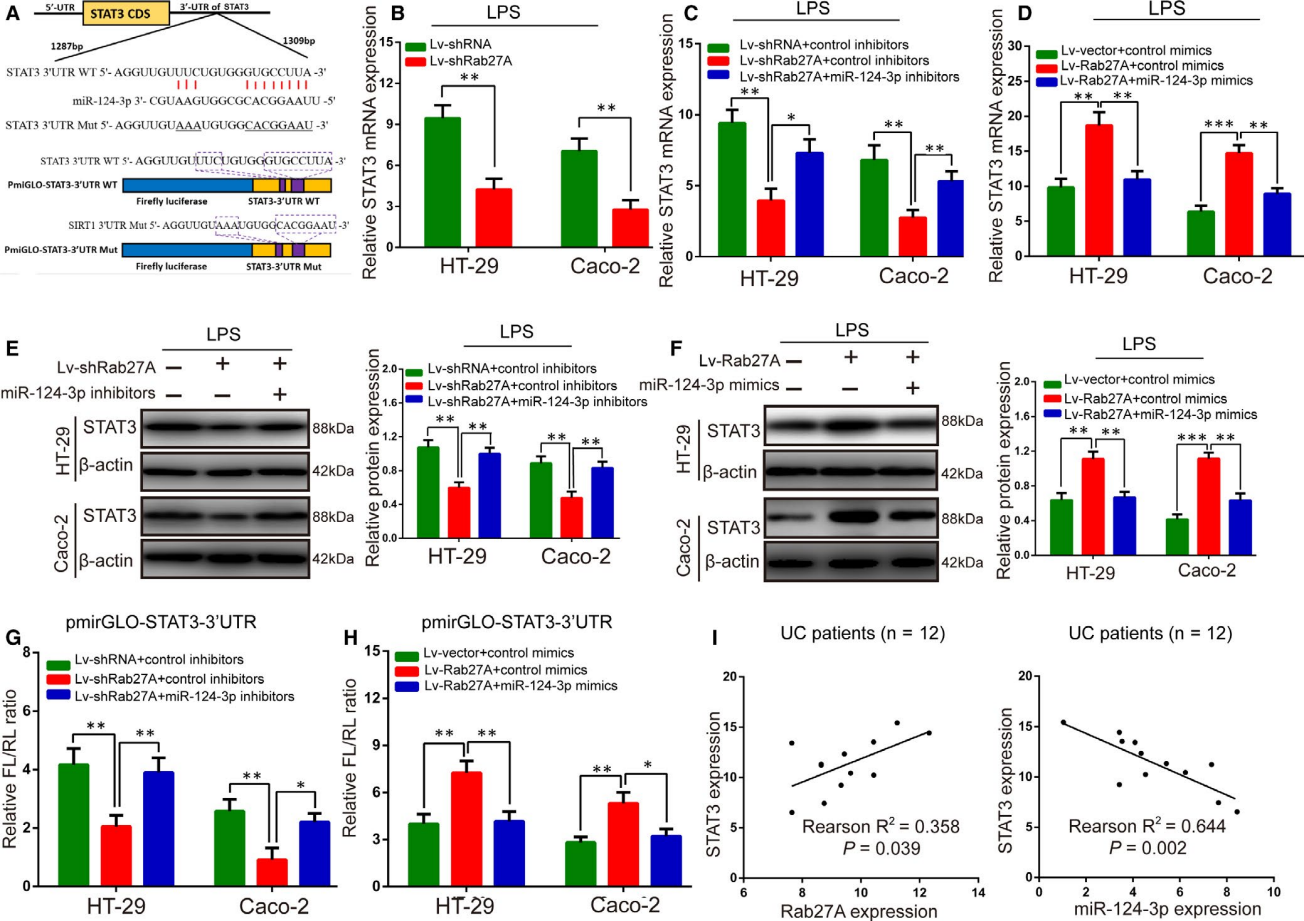
Rab27A regulates STAT3 expression by binding with miR‐124‐3p. A: The miR‐124‐3p target site in the STAT3 3’‐UTR was predicted, and the mutant site of the STAT3 3’‐UTR is also shown. B: The STAT3 mRNA expression level in the presence of Lv‐shRab27A or Lv‐shRNA was detected by RT‐qPCR. C, E: The mRNA and protein expression levels of STAT3 in colonic inflammatory cells transfected with Lv‐Rab27A or cotransfected with Lv‐Rab27A and miR‐124‐3p inhibitors were detected by RT‐qPCR (C) and Western blotting (D) assays. D, F: The mRNA and protein expression levels of STAT3 after transfection with Lv‐Rab27A or cotransfection with Lv‐Rab27A and miR‐124‐3p mimics were detected by RT‐qPCR (D) and Western blotting (F). G: Luciferase activity was detected in colonic inflammatory cells transfected with Lv‐Rab27A or cotransfected with Lv‐Rab27A and miR‐124‐3p inhibitors. H: Luciferase activity was detected after transfection with Lv‐Rab27A or cotransfection with Lv‐Rab27A and miR‐124‐3p mimics. I: STAT3 mRNA expression relationship with Rab27A or miR‐124‐3p levels in UC tissues. Data are presented as means ± SD of three independent experiments. *P < .05, **P < .01, ***P < .001

Increasing evidence has indicated that STAT3 promotes UC progression, and miR‐124‐3p could target the STAT3 3'‐UTR,^20^, ^21^ which was confirmed in inflammatory colonic cells in this study (Figure 6A and Figure S4). miR‐124‐3p exerts its UC progression effects by regulating STAT3 expression in IECs (Figure S5). Therefore, we have been suggested that Rab27A regulates STAT3 expression by binding with miR‐124‐3p. First, we found that Lv‐shRab27A could decrease STAT3 mRNA levels in LPS‑treated IECs (Figure 6B). RT‐qPCR and Western blotting revealed that STAT3 mRNA and protein expression levels induced by Lv‐shRab27A down‐regulation were reversed after the introduction of miR‐124‐3p mimics (Figure 6C,E). Likewise, the inhibitory role of Lv‐Rab27A on STAT3 expression was counteracted via cotransfection with miR‐124‐3p mimics (Figure 6D,F). Moreover, Lv‐shRab27A reduced the luciferase activity of the pmirGLO‐STAT3 3'‐UTR, and this alleviation was restored by miR‐124‐3p inhibitors (Figure 6G). Reciprocally, Lv‐Rab27A accumulated the luciferase activity of the pmirGLO‐STAT3 3'‐UTR, while miR‐124‐3p mimics abolished the above up‐regulation (Figure 6H). Finally, we detected that the STAT3 mRNA expression was significantly positively correlated with Rab27A mRNA expression (*P* = 0.039) and markedly negatively correlated with miR‐124‐3p expression (*P* = 0.002) in UC tissues (Figure 6I). The above results collectively illustrate that the Rab27A 3’‐UTR could bind to miR‐124‐3p to elevate STAT3 expression in UC patients.

### 3.7 Rab27A promoted UC progression via the STAT3/RelA signalling pathway

**FIGURE 7:**
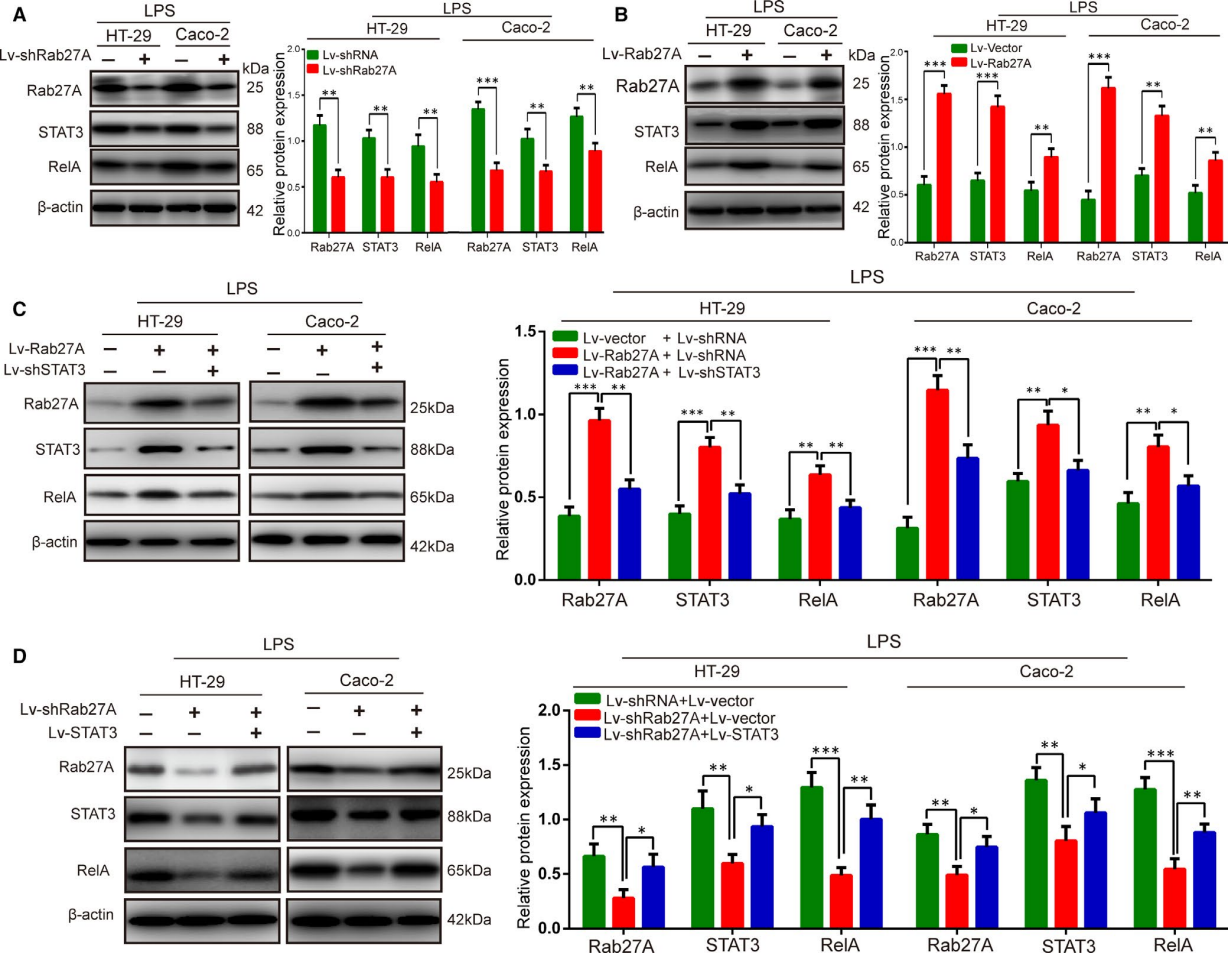
Rab27A promotes UC progression via the STAT3/RelA signalling pathway. A‐B: The expression of the Rab27A, STAT3 and RelA proteins was determined in colonic inflammatory cells transfected with Lv‐shRab27A (A) or Lv‐Rab27A (B). C: The expression of the Rab27A, STAT3 and RelA proteins was determined in colonic inflammatory cells transfected with Lv‐Rab27A or cotransfected with Lv‐Rab27A and Lv‐shSTAT3. D: The expression of the Rab27A, STAT3 and RelA proteins was determined in colonic inflammatory cells transfected with Lv‐shRab27A or cotransfected with Lv‐shRab27A and Lv‐STAT3. Data are presented as means ± SD of three independent experiments. *P < .05, **P < .01, ***P < .001

It is reported that the STAT3/RelA signalling pathway promotes intracellular ROS production to aggravate disease progression in UC.^22^, ^23^ Thus, to further illustrate the potential mechanism involved in Rab27A‐associated exacerbated progression of UC, we determined the expression levels of the STAT3/RelA signalling pathway in LPS‑treated IECs. Western blotting analysis showed that the STAT3 and RelA expression levels were significantly reduced when Rab27A was knocked down (Figure 7A). Moreover, overexpression of Rab27A caused the opposite results (Figure 7B). In addition, Lv‐shSTAT3 treatment in LPS‐IECs interfered with the increase in STAT3 and RelA expression caused by Lv‐Rab27A (Figure 7C). Lv‐STAT3 reversed the effect of Lv‐shRab27A, which alleviated STAT3 and RelA expression (Figure 7D). Therefore, these data indicate that Rab27A may promote UC progression via the STAT3/RelA signalling pathway.

### 3.8 Rab27A promoted ulcerative colitis progression in vivo by regulating the miR‐124‐3p/STAT3/RelA pathway

**FIGURE 8:**
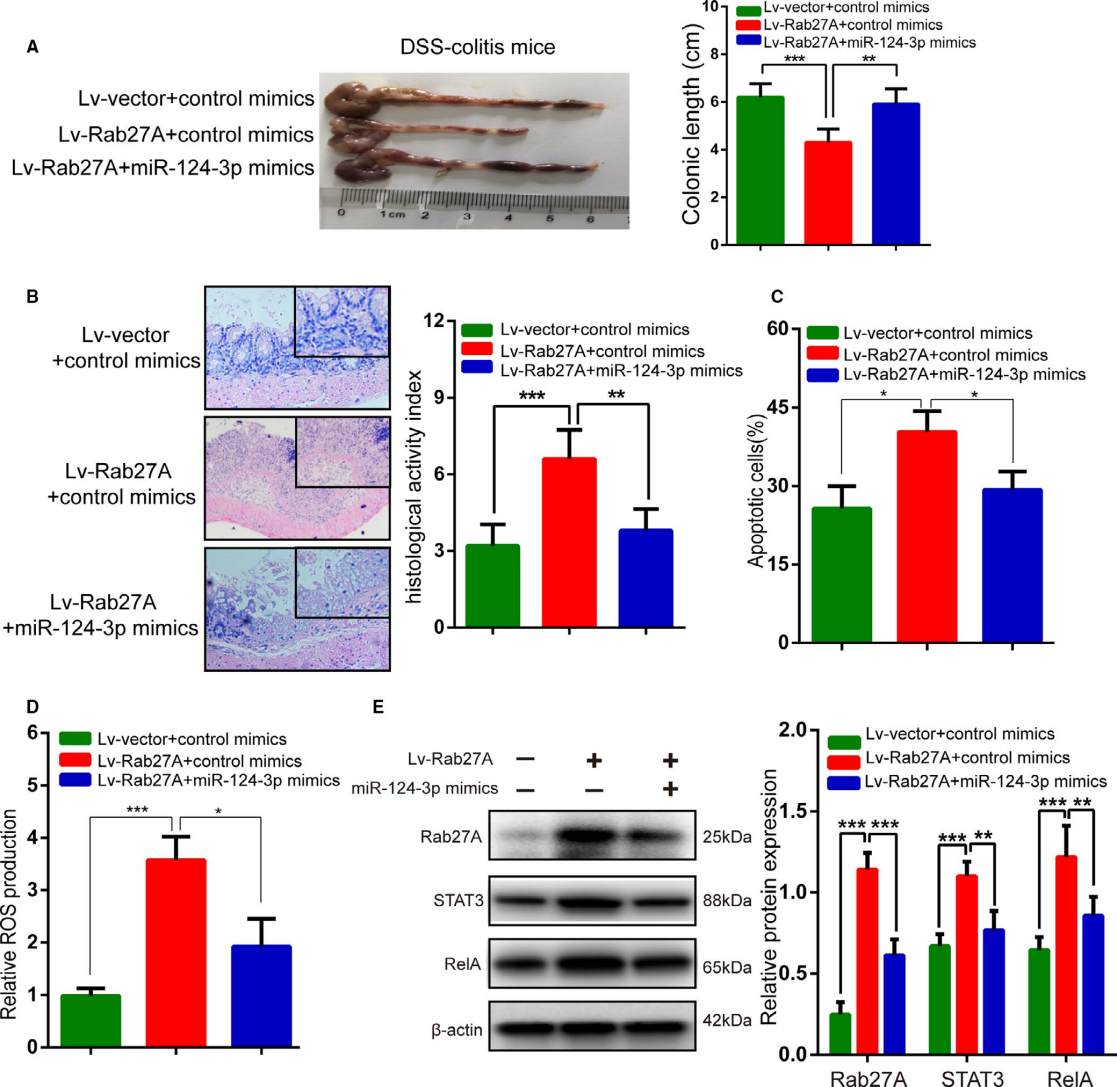
Rab27A promotes UC progression in vivo by regulating the miR‐124‐3p/STAT3/RelA pathway. A‐C: The colonic length (A) and histological score (B‐C) by H&E staining were measured in DSS‐induced colitis mice transfected with Lv‐Rab27A or cotransfected with Lv‐Rab27A and miR‐124‐3p mimics. C‐D: Cellular apoptosis (C) and ROS production (D) were measured in DSS‐induced colitis mice transfected with Lv‐Rab27A or cotransfected with Lv‐Rab27A and miR‐124‐3p mimics. E: The expression of the Rab27A, STAT3 and RelA proteins was determined in DSS‐induced colitis mice transfected with Lv‐Rab27A or cotransfected with Lv‐Rab27A and miR‐124‐3p mimics. Data are presented as means ± SD. **P < .01, ***P < .001

Finally, we sought to illustrate whether miR‐124‐3p could regulate the inflammatory process by targeting Rab27A 3'‐UTR in animal experiments. One group was administered miR‐124‐3p mimics through intracolonic administration, while another group was treated with miR‐124‐3p mimics + Lv‐Rab27A in DSS‐induced mouse colitis. As expected, miR‐124‐3p mimics could interfere with the enhanced inflammatory processes, including the shorter colonic length (Figure 8A), and worse inflammatory cell infiltration (Figure 8B) caused by Lv‐Rab27A. What's more, miR‐124‐3p mimics could also restrict the increased cellular apoptosis (Figure 8C) and ROS production (Figure 8D) caused by Lv‐Rab27A in inflammatory colonic cells of animal experiments. Furthermore, Western blotting assays revealed that Lv‐Rab27A promoted the expression of the STAT3/RelA signalling pathway, and miR‐124‐3p mimics reversed these effects (Figure 8E). Together, these results demonstrated that Rab27A plays a crucial role in ulcerative colitis progression through regulating the miR‐124‐3p/STAT3/RelA pathway.

## 4 DISCUSSION

**FIGURE 9:**
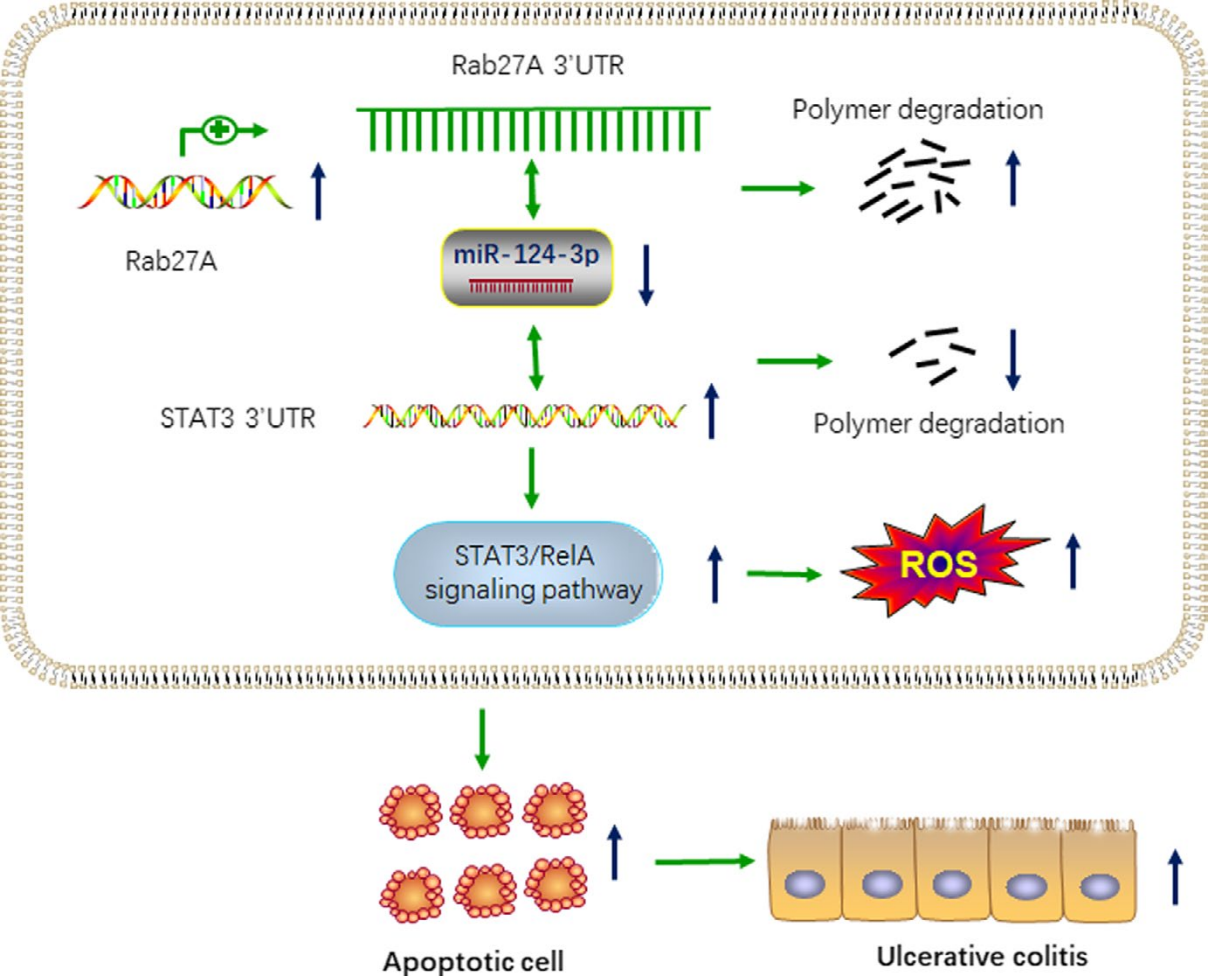
Rab27A promotes ROS production and apoptosis via suppressing miR‐124‐3p and increasing STAT3/RelA pathway in inflammatory colonic cells

In this current study, we illustrated the function and mechanism of Rab27A in inflammatory colonic cells and found that Rab27A mRNA and protein were frequently up‐regulated in UC tissues and DSS‐induced mouse model. Our findings demonstrated that knockdown of Rab27A reduced apoptosis and ROS production in colonic epithelial cells. Furthermore, we clarified that Rab27A stimulated the STAT3/RelA signalling pathway by binding with miR‐124‐3p to promote the progression of ulcerative colitis (Figure 9).

In previous studies, Rab27A was shown to regulate tumour cellular proliferation and apoptosis in a number of malignant tumours, such as lung cancer,^24^ pancreatic carcinoma^25^ and colorectal cancer.^26^, ^27^ Moreover, Rab27A regulates inflammatory responses and consequently contributes to neutrophil functions.^28^, ^29^, ^30^ In this study, Rab27A mRNA and protein were highly expressed in inflammatory colonic mucosa tissues of UC patient and DSS‐induced mice, consistent with the studies of Xu et al^14^ The ROS production and apoptosis of epithelial cells are strongly related to UC pathogenesis. A number of studies have suggested that abnormal apoptosis and ROS in IECs could result from increased cytokine production, such as that of TNF‐α, INF‐γ and IL‐6.^30^, ^31^ From the previous study, Qiao et al reported that LPS could induce cell inflammatory response in ulcerative colitis.^32^ Therefore, we used LPS to induce inflammatory injury in HT‐29 and Caco‐2 cells, and found that LPS treatment significantly promoted the expression of inflammatory factors in colonic cells. In the present study, knockdown of Rab27A significantly reduced apoptosis and ROS production in LPS‑treated colonic cells.

Most of the existing miRNA research has focused on the potential influence of miRNAs in carcinoma development,^33^ and much less is known about how miRNAs affect UC. Elucidating the role of miRNAs in UC embodies a new frontier to deepen our understanding of the pathogenesis of diseases and to develop new diagnostic and therapeutic tools.^7^, ^8^ Previous data on miR‐124‐3p mainly have focused mainly on its function in various forms of cancer, such as colorectal cancer, gastric carcinoma and hepatocellular cancer.^34^, ^35^ A recent study illustrated that miR‐124‐3p plays a role in immune function and is involved in the regulation of various inflammatory reactions.^21^ Koukos and others illustrated that down‐regulated miR‐124‐3p participates in the colonic epithelial cell inflammatory response and is implicated in UC pathogenesis.^36^, ^37^ Tang et al illustrated miR‐124‐3p inhibited osteoclastogenic differentiation of bone marrow monocytes by directly suppressing Rab27a expression.^13^ Wu et al found Rab27a could promote proliferation and invasion, and suppress cell apoptosis by targeting miR‐124‐3p in glioma cell.^38^ In our research, miR‐124‐3p expression was notably increased by treatment with Lv‐shRab27A and significantly repressed by treatment with Lv‐Rab27A in inflammatory colonic cells. What's more, miR‐124‐3p was found to possess a binding site for the Rab27A 3'UTR, and Rab27A 3’‐UTR was a direct target of miR‐124‐3p by luciferase reporter assay. Furthermore, the down‐regulation of Rab27A greatly inhibited the cellular apoptosis or ROS production, and miR‐124‐3p inhibitors could counteract these effects, and miR‐124‐3p mimics promoted the effect of Lv‐shRab27A. In addition, up‐regulation of Rab27A increased the cellular apoptosis and ROS production, and miR‐124‐3p mimics reversed these effects induced by Lv‐Rab27A. Thus, we concluded that Rab27A stimulates ulcerative colitis progression through binding miR‐124‐3p. However, elucidating the exact mechanism that Rab27A directly or indirectly by targeting miR‐124 regulate ROS production and apoptosis in UC development will require further studies.

STAT3 abnormal expression is associated with colonic inflammation and activated by various growth factors and cytokines.^20^, ^39^ STAT3/RelA signalling pathway is identified as a classical pro‐inflammatory pathway because of the association of RelA with pro‐inflammatory cytokines, chemokines and adhesion molecules.^40^ And it could mediate the intestinal epithelial cell apoptosis and ROS production in ulcerative colitis, which may play a central role in cell fate decision.^41^ Wang et al demonstrated miR‐124‐3p inhibited STAT3 expression by directly targeting its 3‐UTR to suppressed apoptosis and promoted cell cycle progression, migration and proliferation.^42^ Chen et al illustrated miR‐124‐3p regulated RelA signalling pathway, then increasing the inflammatory response and decreasing the As2O3 injury process of cardiomyocytes.^43^ In the colitis model, inhibition of STAT3 and RelA expression could ameliorate colonic inflammatory damage by down‐regulating pro‐inflammatory cytokines.^44^ In our study, Rab27A knockdown reduced cellular apoptosis and ROS production in LPS‑induced colonic cells, while cotransfection with miR‐124‐3p inhibitors negated these responses. Then, we demonstrated that Rab27A competitively binds miRNA‐124‐3p to regulate the STAT3/RelA signalling pathway in LPS‑induced colonic cells by dual‐luciferase reporter assay and Western blotting.

In conclusion, the significant increase in Rab27A mRNA and protein identified in this study make it a potential candidate as a biomarker for UC in the future. In addition to the insights into the pathology of this disease, we found a new pathway in the mechanism of UC: Rab27A, by regulating miR‐124‐3p, can activate the STAT3/RelA signalling pathway, which may provide novel therapeutic approaches with great impact in ulcerative colitis.

## CONFLICT OF INTEREST

The authors declare no competing financial interests.

## AUTHOR CONTRIBUTIONS

Yang Luo and Min‐Hao Yu: Cytology experiments, data analysis and manuscript writing. Ya‐Ru Yan: Bioinformatics analysis. Yong Zhou and Shao‐Lan Qin: Intracolonic administration. Yi‐Zhou Huang and Jun Qin: Immunohistochemistry staining and Western blotting. Ming Zhong: Idea for the project conception and manuscript editing.

## Supporting information

Fig S1

Fig S2

Fig S3

Fig S4

Fig S5

Table S1

Table S2

## Data Availability

Data sets used and analysed during the current study are available from the corresponding author on reasonable request.
