# Supplementary figures and images for: Rab27A promotes cellular apoptosis and ROS production by regulating the miRNA‐124‐3p/STAT3/RelA signalling pathway in ulcerative colitis
Source: J Cell Mol Med. 2020 Aug 20;24(19):11330–42. doi: 10.1111/jcmm.15726 (PMC7576264; doi:10.1111/jcmm.15726)

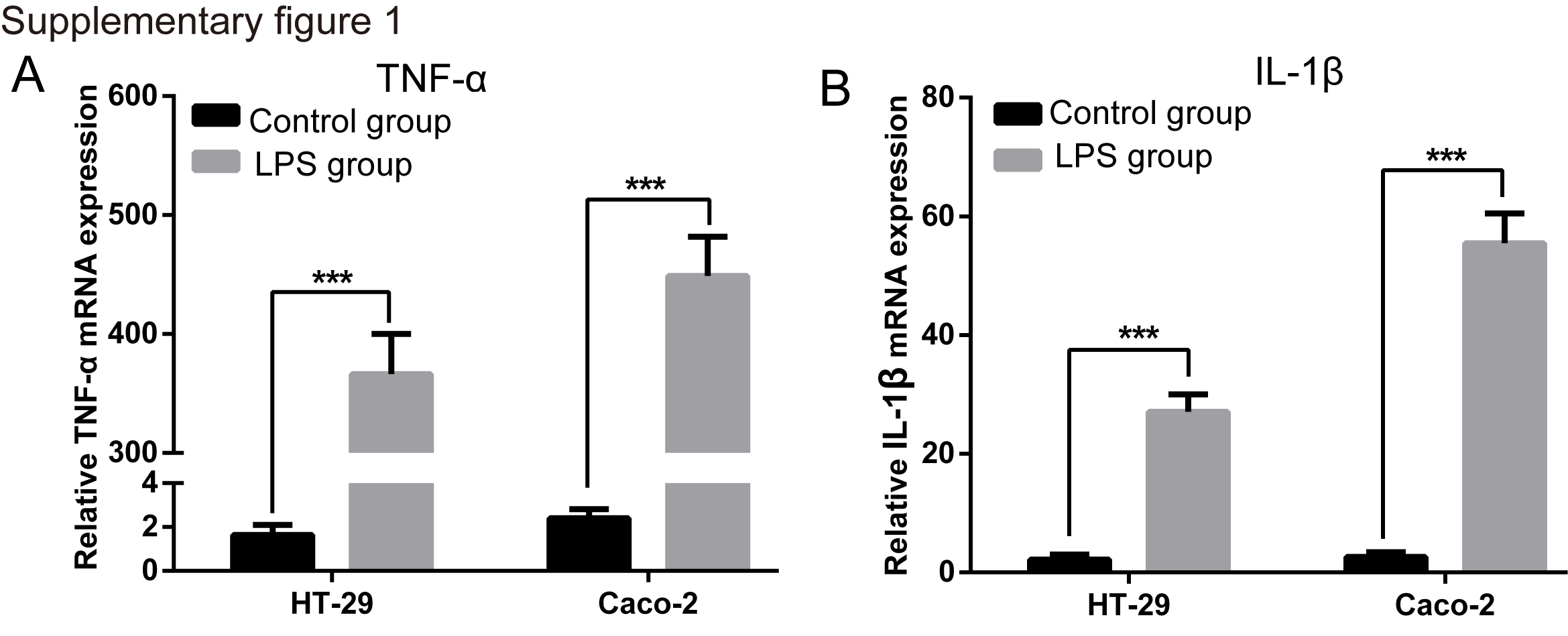

Supplement: Supplementary file 1 — Fig S1 [file JCMM-24-11330-s001.tif]

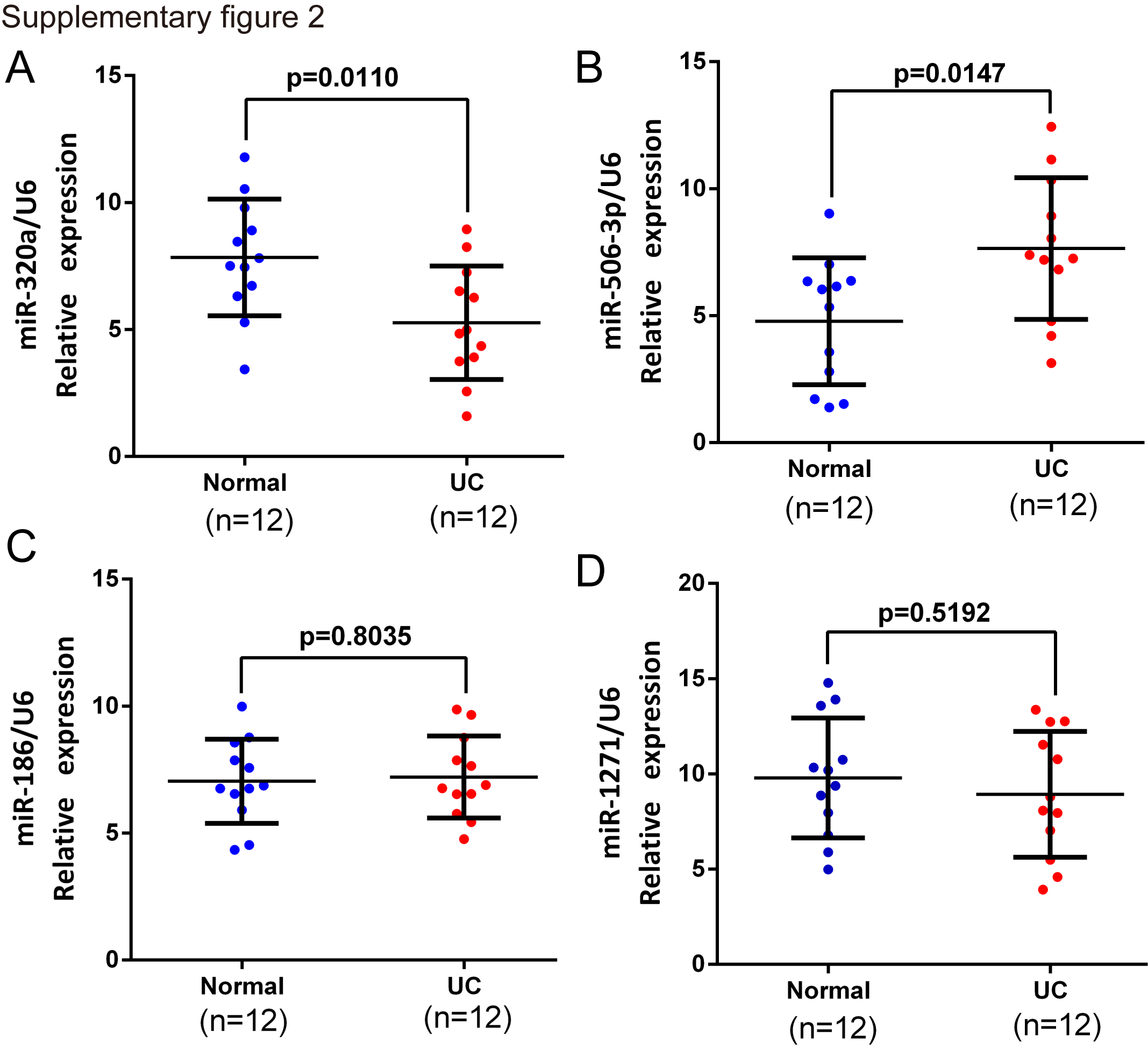

Supplement: Supplementary file 2 — Fig S2 [file JCMM-24-11330-s002.tif]

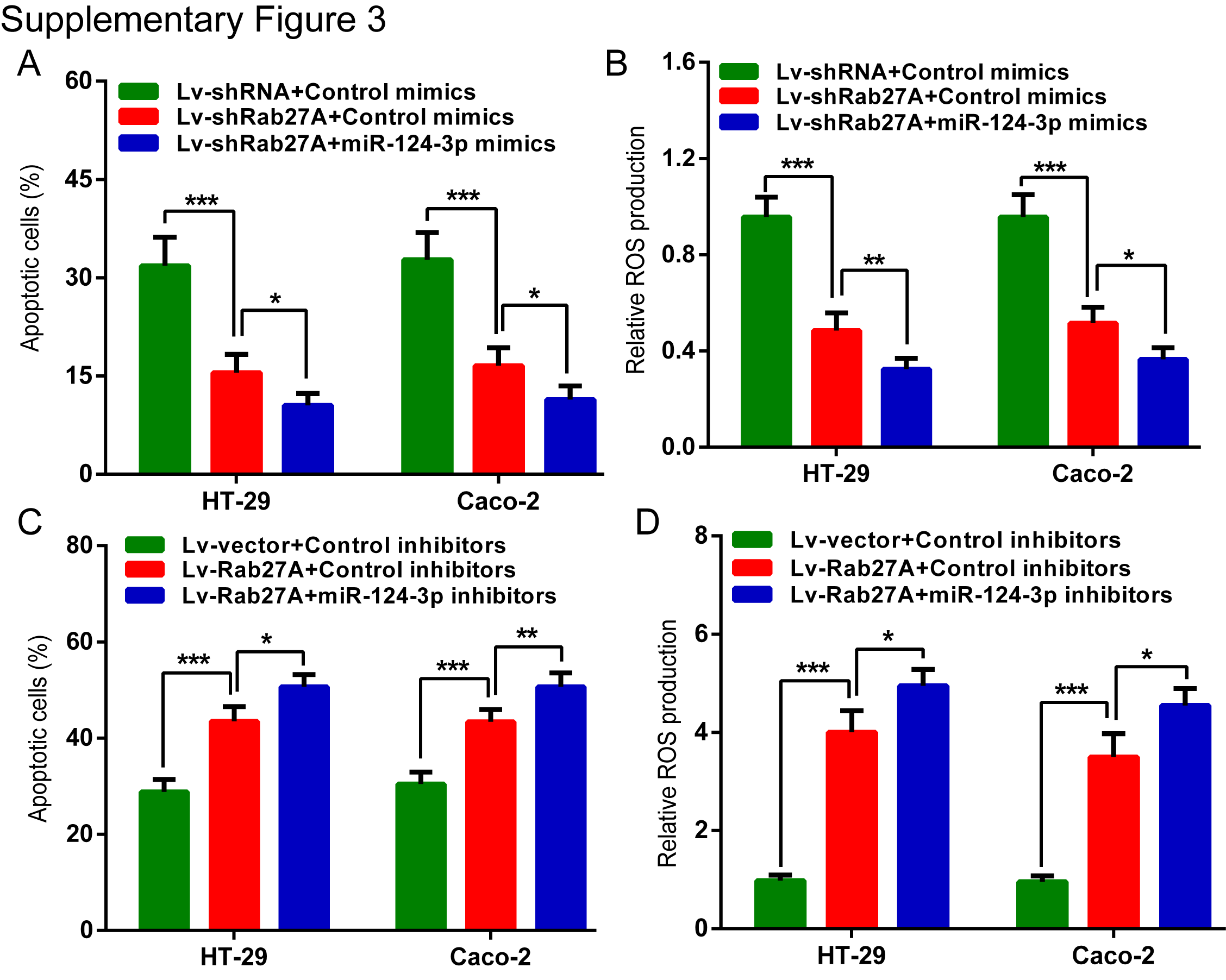

Supplement: Supplementary file 3 — Fig S3 [file JCMM-24-11330-s003.tif]

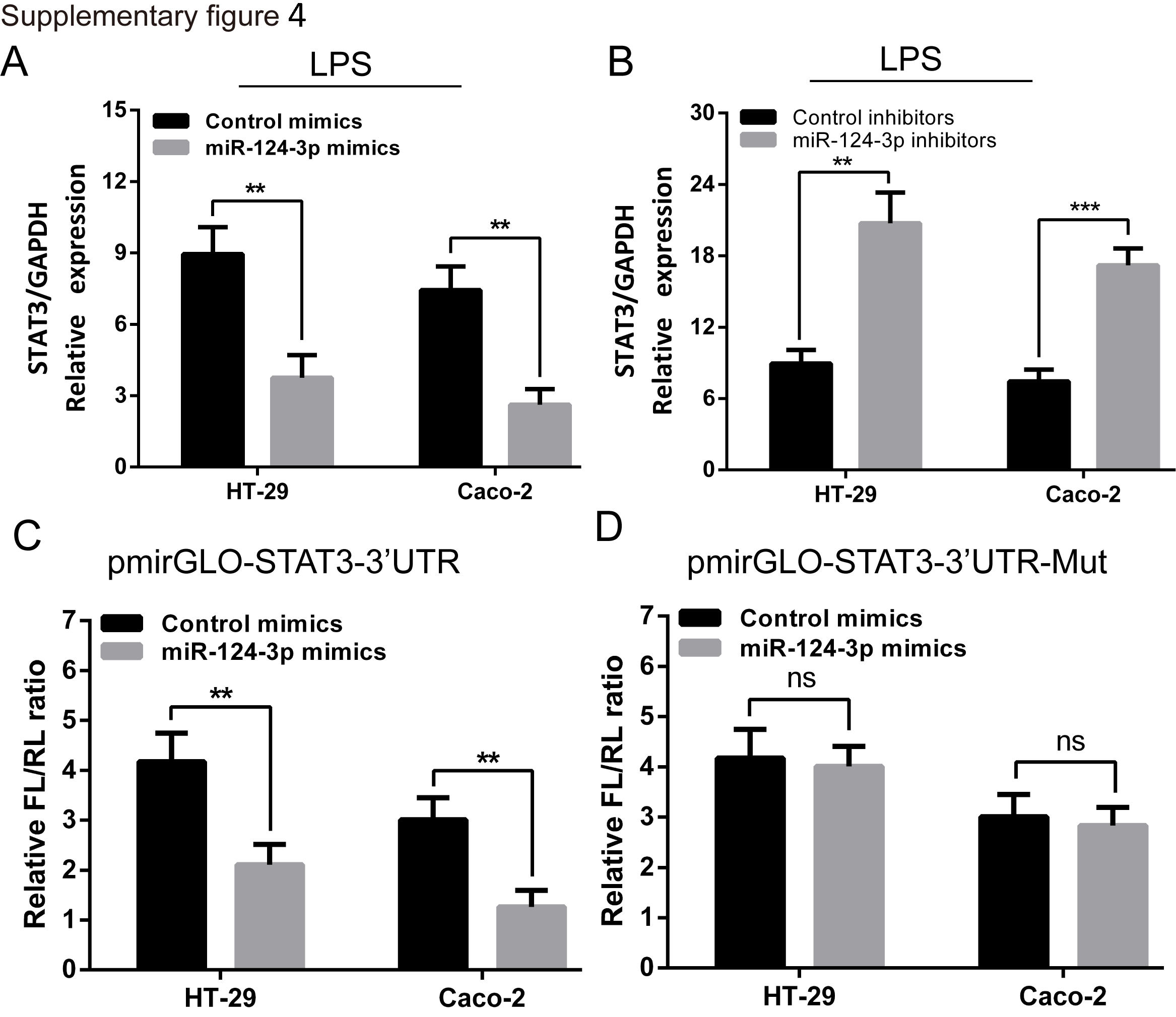

Supplement: Supplementary file 4 — Fig S4 [file JCMM-24-11330-s004.tif]

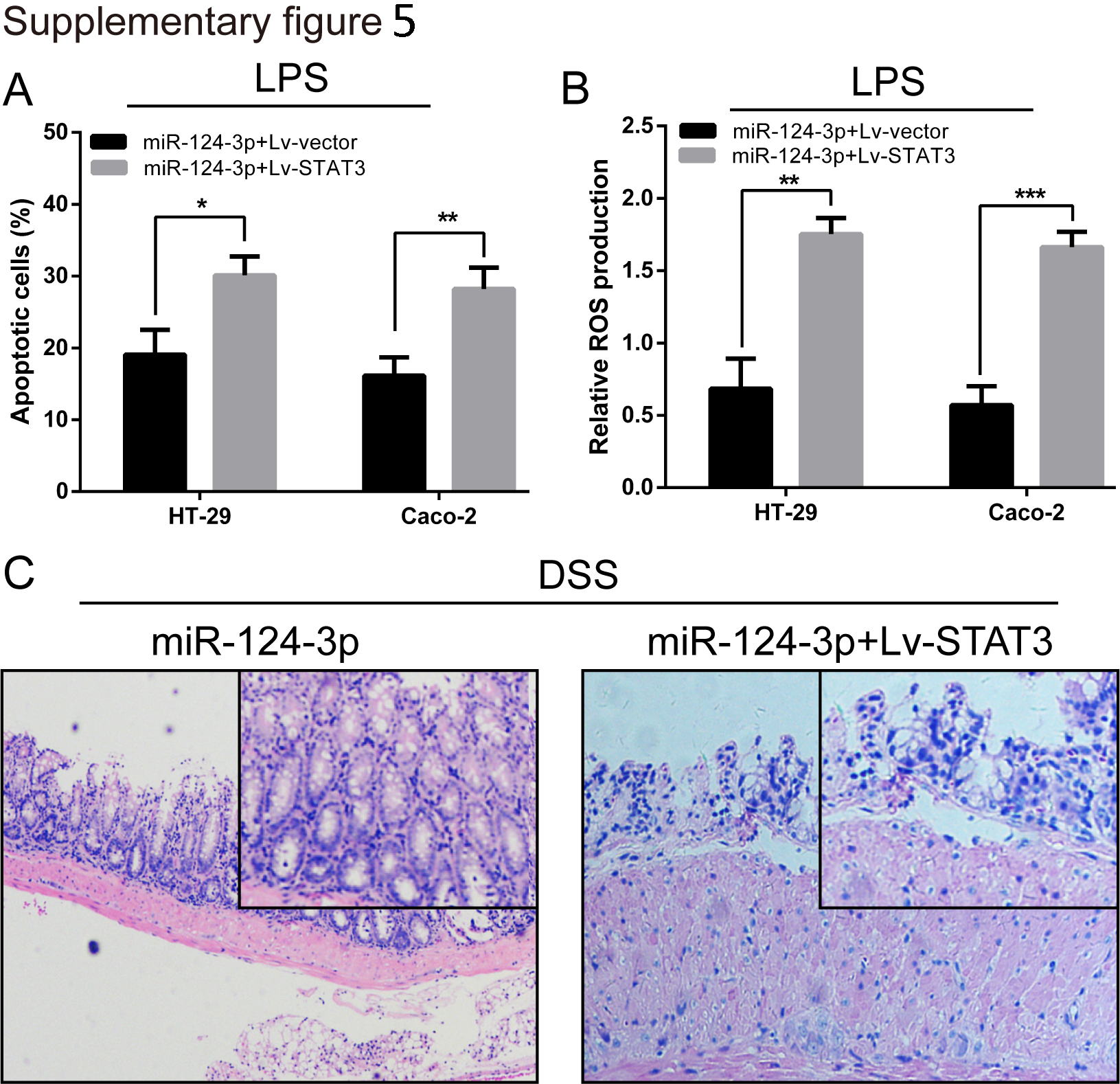

Supplement: Supplementary file 5 — Fig S5 [file JCMM-24-11330-s005.tif]
