# Supplementary material for: Rab27A promotes cellular apoptosis and ROS production by regulating the miRNA‐124‐3p/STAT3/RelA signalling pathway in ulcerative colitis
Source: J Cell Mol Med. 2020 Aug 20;24(19):11330–42. doi: 10.1111/jcmm.15726 (PMC7576264; doi:10.1111/jcmm.15726)
Supplement: Supplementary file 6 — Table S1 [file JCMM-24-11330-s006.docx]

Supplementary table 1. The sequences of mimics and inhibitors

| **Gene** | **Sequences** |
| --- | --- |
| miR-124-3p mimics | 5′-UAAGGCACGCGGUGAAUGCC-3′ |
| miR-96-5p mimics | 5′-UUUGGCACUAGCACAUUUUUGCU-3′ |
| Control mimics | 5′-UUUGUACUACACAAAAGUACUG-3′ |
| miR-124-3p inhibitors | 5′-CGUGUUCACAGCGGACCUUGAU-3′ |
| miR-96-5p inhibitors | 5′-AGCAAAAAUGUGCUAGUGCCAAA-3′ |
| Control inhibitors | 5′-CAGUACUUUUGUGUAGUACAAA-3′ |
