# Supplementary material for: Rab27A promotes cellular apoptosis and ROS production by regulating the miRNA‐124‐3p/STAT3/RelA signalling pathway in ulcerative colitis
Source: J Cell Mol Med. 2020 Aug 20;24(19):11330–42. doi: 10.1111/jcmm.15726 (PMC7576264; doi:10.1111/jcmm.15726)
Supplement: Supplementary file 7 — Table S2 [file JCMM-24-11330-s007.doc]

**Supplementary Table 2. The sequence of wild type and mutant 3’UTR of Rab27a**

| **Gene** | **Sequence** |
| --- | --- |
| wild-type Rab27A 3’-UTR | 5’^_^gaagucaaguaagcgacauaguaguucagguggcccaugccugggaucuucucuaugauugauacauggcacagugagagauuaaugggcauuguguacaaauugcuucucaccauccccauuagaccuacgaauaaagcauccgguucuaaaauuaauuuguugcagcuuuguaaauauuucuuuaagauucagccugagaguuaggagaaauauuucagagccaaaagugccuuauacaaccuuagccuauuauaguaaaucauucaaggauucagaauuuugcagucacagaagaguguauuuauuauguagaaugaaugaggguacugucaccugccuuaauguagguaggcccagagucuuacauuuaagaucuuacaugcaguuauaaaaccgccacagucuucaauccagauuugaagacucaugccauaggugacauucuaaaauaccauuaaagccacuuaaauguuaaauaagaauauacaugcacaucagcucaaugucuuugaguauuaauuuuauguaagcauucuauuuaacaugaauauaggacaaaucauggcuauaucuauagaccuuggauaaacuggauugaccaauuauacacucacggugacuuuuuuauuggugggaaggggauugggguggggcaggcuggcuuaauguaauaugagcaaccaaagugggacuucugucuccccgcuauauucccauugcucugaaugguugauugaagggucagggaacuagauuuuauggcuuuaguucacugugauuguacauuuauacuuggccuaugugcuggccgcaccugaacauagcuggugcuuaugccgaguuauuugcgaugaguaaauauuuaguuucuuuuucuucauauuuauaauguugaucuggcauccucaggcugcagcuuuauuagcuuauaacuuacucaucucuaucuuuaccagcaggcucuguauuguugauauuugcaacuuguuuugcuuuuccauugguggaauugaaauaauuaguuuuuaauuacauaagaugccuguuugcuauuugguggaagauagauguucauauugaagcagucacauuuguacuguaguucaauaaaagaaaaaugaaguauucuguagccuauauuuuucauagagcucaugagcauuuacuguacuugcugggucuugccaagaucauuuauuccgcugcauugccaaagugucuucauaccaaauuaaaggugguuuuaauauauguuucauggaaguuguuuauaaaauucaaagguauuucauuuaggugaaaagucuuauuuauuaaagugguuugaauaaaguagaucaaaacuuccagagaucuuaauggcuauauaggaagaaauaucacucaccauaauuuaaauaaagaauaaaaauacuuguauuuugugguggcaaauguuugguagaacuguaauuagaaaaauacaaguauauuugcgugaugguuacacuagaagcccagacuuuacgacuacacaauauauucauguaucuaaacuguacuuguacccccuaaauuuauuuuuaaaaaaggaaaaauaaaaguaucaugaaaaaaccuauuuuuuuuuccacuguccuuccacuacucccauaacaaacuuauccaugguugguaaaauuuuacauauuucuauccuugaaaugaaggcuucuuuuaaauuccaaagaagucauggaggccugugcauuugaauuguauaugcuagugaggaaaagauuuagacauuucaagagcaggguuggccaggcgcgguggcucacaccuguaaucccagcacuuugggaggccgaggagggcggaucacgaggucaggagaucgagaccauccuggcuaacacagugaaaccccaucucuacuaaaaaaaaaaaaaaaaaaaaaaugggcugggcgugguggugggcaccuguagucccagcuacucaggaggcugaggcaggagaauggcgugaaccugggaggcggagcuugcagugagccgagauugcgccacugcacuccagccugggcgacagaggagacucugucucaaaaaaaaaaaaaaaaaaaaagagcagggucauaaucacacagcagugccuuauauaguugccauaagacuucagugcaguacaacauaauuuuacagcuacauaucagggcauauucuauaugguguauuuguguuagaauaacacauuaaaugucuuuaaacauaaaaauaagaauguuugcauguuucaguuuucaagaaccaaaugaguaauuagcuauagauuccacuggccuuaaacauacaauuaaguguauacaugauauagugcacacacaaaagccaccuuuaauuauugaaauaaccuguauucuuuuuggaaaucauuuaaguuugguauugaaguacuauauuuuuugugcaucaauguauuuuucuauuuacaagccuauguaaaagugaaguguaucuucagugaaccaugugccaauuaagcuguaauaaaaaaguggucuagucugucaaaaaaaaaaaaaaaaaa -3’ |
| Mutant Rab27A 3’-UTR | 5’_gaagucaaguaagcgacauaguaguucagguggcccaugccugggaucuucucuaugauugauacauggcacagugagagauuaaugggcauuguguacaaauugcuucucaccauccccauuagaccuacgaauaaagcauccgguucuaaaauuaauuuguugcagcuuuguaaauauuucuuuaagauucagccugagaguuaggagaaauauuucagagccaaaagugccuuauacaaccuuagccuauuauaguaaaucauucaaggauucagaauuuugcagucacagaagaguguauuuauuauguagaaugaaugaggguacugauuuuauaaaauuuguagguaggcccagagucuuacauuuaagaucuuacaugcaguuauaaaaccgccacagucuucaauccagauuugaagacucaugccauaggugacauucuaaaauaccauuaaagccacuuaaauguuaaauaagaauauacaugcacaucagcucaaugucuuugaguauuaauuuuauguaagcauucuauuuaacaugaauauaggacaaaucauggcuauaucuauagaccuuggauaaacuggauugaccaauuauacacucacggugacuuuuuuauuggugggaaggggauugggguggggcaggcuggcuuaauguaauaugagcaaccaaagugggacuucugucuccccgcuauauucccauugcucugaaugguugauugaagggucagggaacuagauuuuauggcuuuaguucacugugauuguacauuuauacuuggccuaugugcuggccgcaccugaacauagcuggugcuuaugccgaguuauuugcgaugaguaaauauuuaguuucuuuuucuucauauuuauaauguugaucuggcauccucaggcugcagcuuuauuagcuuauaacuuacucaucucuaucuuuaccagcaggcucuguauuguugauauuugcaacuuguuuugcuuuuccauugguggaauugaaauaauuaguuuuuaauuacauaagaugccuguuugcuauuugguggaagauagauguucauauugaagcagucacauuuguacuguaguucaauaaaagaaaaaugaaguauucuguagccuauauuuuucauagagcucaugagcauuuacuguacuugcugggucuugccaagaucauuuauuccgcugcauugccaaagugucuucauaccaaauuaaaggugguuuuaauauauguuucauggaaguuguuuauaaaauucaaagguauuucauuuaggugaaaagucuuauuuauuaaagugguuugaauaaaguagaucaaaacuuccagagaucuuaauggcuauauaggaagaaauaucacucaccauaauuuaaauaaagaauaaaaauacuuguauuuugugguggcaaauguuugguagaacuguaauuagaaaaauacaaguauauuugcgugaugguuacacuagaagcccagacuuuacgacuacacaauauauucauguaucuaaacuguacuuguacccccuaaauuuauuuuuaaaaaaggaaaaauaaaaguaucaugaaaaaaccuauuuuuuuuuccacuguccuuccacuacucccauaacaaacuuauccaugguugguaaaauuuuacauauuucuauccuugaaaugaaggcuucuuuuaaauuccaaagaagucauggaggccugugcauuugaauuguauaugcuagugaggaaaagauuuagacauuucaagagcaggguuggccaggcgcgguggcucacaccuguaaucccagcacuuugggaggccgaggagggcggaucacgaggucaggagaucgagaccauccuggcuaacacagugaaaccccaucucuacuaaaaaaaaaaaaaaaaaaaaaaugggcugggcgugguggugggcaccuguagucccagcuacucaggaggcugaggcaggagaauggcgugaaccugggaggcggagcuugcagugagccgagauugcgccacugcacuccagccugggcgacagaggagacucugucucaaaaaaaaaaaaaaaaaaaaagagcagggucauaaucacacagcagugccuuauauaguugccauaagacuucagugcaguacaacauaauuuuacagcuacauaucagggcauauucuauaugguguauuuguguuagaauaacacauuaaaugucuuuaaacauaaaaauaagaauguuugcauguuucaguuuucaagaaccaaaugaguaauuagcuauagauuccacuggccuuaaacauacaauuaaguguauacaugauauagugcacacacaaaagccaccuuuaauuauugaaauaaccuguauucuuuuuggaaaucauuuaaguuugguauugaaguacuauauuuuuugugcaucaauguauuuuucuauuuacaagccuauguaaaagugaaguguaucuucagugaaccaugugccaauuaagcuguaauaaaaaaguggucuagucugucaaaaaaaaaaaaaaaaaa -3’ |
